# Periodontal risk assessment and associated factors in young adults: a cross-sectional study

**DOI:** 10.1186/s12903-026-08427-y

**Published:** 2026-04-28

**Authors:** Resül Çolak, Merve Küçükoğlu Çolak, Sedef Ercan

**Affiliations:** Department of Periodontics, Faculty of Dentistry, Zonguldak Bülent Ecevit University, Zonguldak, Esenköy/Kozlu 67600 Türkiye

**Keywords:** Risk assessment, Risk evaluation, Periodontal disease progression, Risk factors

## Abstract

**Background and aim:**

Periodontal diseases are common chronic inflammatory conditions with a multifactorial etiology among young adults. Periodontal Risk Assessment (PRA) enables the systematic identification of individual disease risk, thereby facilitating early intervention and the implementation of preventive strategies. This study aimed to determine PRA risk levels in a young adult population and to investigate the demographic, environmental, and periodontal factors associated with these risk levels.

**Materials and methods:**

A total of 100 volunteer individuals who presented for routine dental examination were included in the study. Demographic, behavioral, and periodontal parameters were recorded. Clinical assessments comprised the plaque index, gingival index, probing depth, bleeding on probing, number of missing teeth, and radiographic bone loss. Periodontal risk levels were classified according to the PRA functional diagram. Statistical significance was set at *p* < 0.05.

**Results:**

Of the participants, 38% were classified as low risk, 46% as moderate risk, and 16% as high risk according to PRA. In the high risk PRA group, males exhibited a 4.30-fold higher periodontal disease risk compared with females, and single individuals demonstrated a higher risk than married participants. Furthermore, increased plaque index, gingival index, and probing depth values were significantly associated with a higher PRA risk.

**Conclusion:**

PRA analyses in young adults showed that a substantial proportion of participants had moderate or high periodontal risk. Male sex, single marital status, elevated plaque and gingival indices, and increased probing depth were associated with higher risk. These findings highlight PRA as a valuable tool for early periodontal risk assessment in young adults.

**Clinical relevance:**

Early identification of periodontal risk in young adults can guide preventive interventions and reduce the risk of disease progression.

**Supplementary Information:**

The online version contains supplementary material available at 10.1186/s12903-026-08427-y.

## Introduction

Periodontal diseases are common chronic inflammatory conditions with a multifactorial etiology that affect more than half of the global population and constitute a substantial public health burden worldwide [1]. Therefore, a standardized classification system is essential for clinicians and researchers to facilitate accurate diagnosis, investigation of the etiology and pathogenesis, and appropriate treatment planning of periodontal diseases. Periodontal disease classifications were first proposed at the 1966 World Workshop in Periodontology and have subsequently been revised several times (1977, 1986, 1999, and most recently in 2017) to reflect advances in scientific knowledge [2]. The 2017 World Workshop, organized by the American Academy of Periodontology (AAP) and the European Federation of Periodontology (EFP), introduced an updated classification system aiming to provide a more comprehensive and clinically applicable framework, particularly suitable for general dental practice, where the majority of periodontal diseases are diagnosed and treated [3]. Gingivitis represents an initial, reversible stage of periodontal disease characterized by inflammation in response to microbial dental plaque accumulation, without accompanying connective tissue or alveolar bone loss. In contrast, periodontitis is a polymicrobial, multifactorial chronic disease characterized by the apical migration of the junctional epithelium along the root surface, leading to the destruction of the periodontal ligament and alveolar bone [4–8].

Periodontal diseases may result in functional impairments, such as tooth loss, increased tooth mobility, hypersensitivity, gingival recession or enlargement, oral malodor, and malocclusion. In advanced stages, periodontal disease is not confined to oral manifestations but is also associated with systemic conditions, including cardiovascular diseases and diabetes mellitus [9]. Importantly, periodontal disease is not limited to older populations; it also has a considerable prevalence among younger individuals. A study conducted in young adults reported periodontal disease in 71.3% of participants [10]. Similarly, a study from Morocco investigating adolescents aged 12–20 years attending public schools demonstrated a high prevalence of aggressive periodontitis. In this young population, smoking and increased periodontal probing depth (PPD ≥ 4 mm), particularly during late adolescence, were identified as significant risk factors for the development of periodontitis in early adulthood. These findings underscore the importance of early diagnosis, risk-based monitoring, and the implementation of preventive strategies in young individuals [11, 12].

Given the multifactorial etiology of periodontal diseases, numerous epidemiological studies have examined the influence of behavioral, genetic, and environmental factors [13–15]. Previous meta-analyses have indicated that oral hygiene practices, tobacco use, genetic susceptibility, and tooth loss are critical determinants of the risk of periodontal disease. However, many studies have focused on isolated risk factors, despite evidence that no single factor alone is sufficient to explain disease development within a multifactorial framework [16–18]. Consequently, the assessment of individualized risk profiles is of paramount importance for the prevention, treatment planning, and long-term management of periodontal disease.

The Periodontal Risk Assessment (PRA) model, developed by Lang and Tonetti in 2003, provides a comprehensive and systematic approach for determining individual periodontal risk by integrating biological and environmental parameters. The model incorporates key biological indicators, such as bleeding on probing (BOP), residual pocket depth, tooth loss, systemic and genetic factors (e.g., diabetes), and age-related alveolar bone loss, alongside environmental factors such as smoking status [19]. While most existing studies have focused on disease diagnosis, progression, and general risk factors [13], investigations addressing individualized risk assessment remain limited in number [20]. Moreover, most available studies have evaluated PRA primarily in the post-treatment maintenance phase, resulting in a paucity of data regarding pretreatment risk profiles [21, 22].

Accordingly, the aim of the present study was to determine periodontal disease risk levels using the PRA model in a young adult population and to evaluate the distribution of PRA risk categories. Additionally, this study investigated the effects of periodontal parameters, as well as demographic and environmental predispositional factors, on different PRA risk groups. It was hypothesized that higher PRA risk scores, in conjunction with specific predispositional factors, would be significantly associated with an increased risk of periodontal disease.

## Materials and methods

### Study design, ethics, and consent

This cross-sectional observational pilot study was conducted in accordance with the STROBE (Strengthening the Reporting of Observational Studies in Epidemiology) guidelines. The study included 100 participants (49 men and 51 women) who attended the Periodontology Clinic of Zonguldak Bülent Ecevit University, Faculty of Dentistry, between January and April 2025 for routine dental examinations or periodontal treatment.

Ethical approval was obtained from the Non-Interventional Clinical Research Ethics Committee of Zonguldak Bülent Ecevit University before the study was initiated (Approval No: 2025/01–20). All procedures were conducted in accordance with the principles of the Declaration of Helsinki, and written informed consent was obtained from all participants before participation.

The participants were included according to the following criteria:

- Adults aged between 18 and 35 years;
- No history of periodontal treatment;
- Non-pregnant and non-lactating women;
- No active orthodontic appliances;
- No use of medications known to affect periodontal tissues, such as cyclosporine A, phenytoin, or calcium channel blockers;
- No diagnosis of dementia or cognitive impairment;
- No history of psychiatric disorders or use of psychotropic medications that could influence anxiety levels.

Participants who did not meet these criteria were excluded from the study.

### Participant characteristics and data collection

**Fig. 1:**
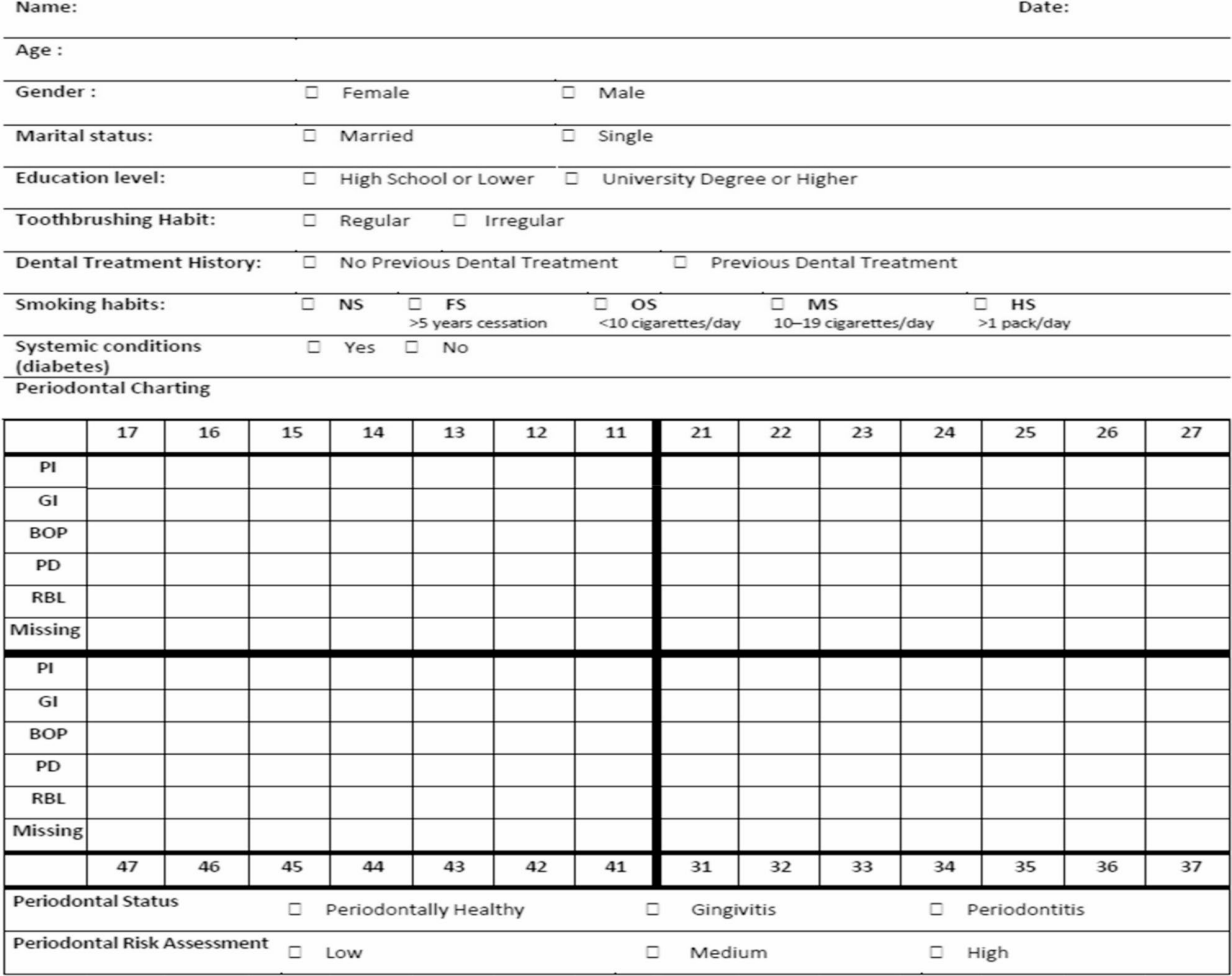
Standardized data collection form used in the study

Demographic and behavioral characteristics, including age, sex, marital status, educational level, toothbrushing habits, dental treatment history, smoking status, and presence of systemic diseases, were recorded using a standardized data collection form (Fig. 1).

Participants were classified according to dental treatment history (no previous dental treatment or previous dental treatment excluding periodontal therapy) [23], educational level (high school or lower vs. university degree or higher) [24], tooth brushing habits (regular vs. irregular) [25], systemic and genetic conditions (present vs. absent), and smoking status as non-smokers (NS), former smokers (FS; cessation > 5 years), occasional smokers (OS; <10 cigarettes/day), moderate smokers (MS; 10–19 cigarettes/day), and heavy smokers (HS; ≥20 cigarettes/day) [19].

### Clinical and radiographic periodontal examination

All periodontal examinations were performed by a single calibrated examiner (S.E.) using a UNC-15 manual periodontal probe (CP-15 UNC, Hu-Friedy^®^, Chicago, IL, USA). Measurements were recorded to the nearest millimeter.

The following periodontal parameters were assessed:

- Plaque Index (PI) [26];
- Gingival Index (GI) [27];
- Probing pocket depth (PPD) [28];
- Bleeding on probing (BOP);
- Number of missing teeth [19];
- Radiographic bone loss (RBL) [29].

Probing pocket depth was defined as the distance from the gingival margin to the base of the periodontal pocket. Clinical attachment loss was determined in teeth exhibiting gingival recession by measuring the distance from the cementoenamel junction to the base of the pocket at the mid-buccal site.

Bleeding on probing was assessed according to the Bleeding Index described by Ainamo and Bay, with bleeding-positive sites recorded as (+) and bleeding-negative sites as (−) [30].

### Periodontal risk assessment and classification

Sociodemographic data and clinical and radiographic periodontal measurements relevant to PRA were incorporated into the PRA functional diagram. Periodontal health status was defined according to the classification proposed by the American Academy of Periodontology (AAP) at the International Workshop on the Classification of Periodontal and Peri-Implant Diseases and Conditions [4].

**Fig. 2:**
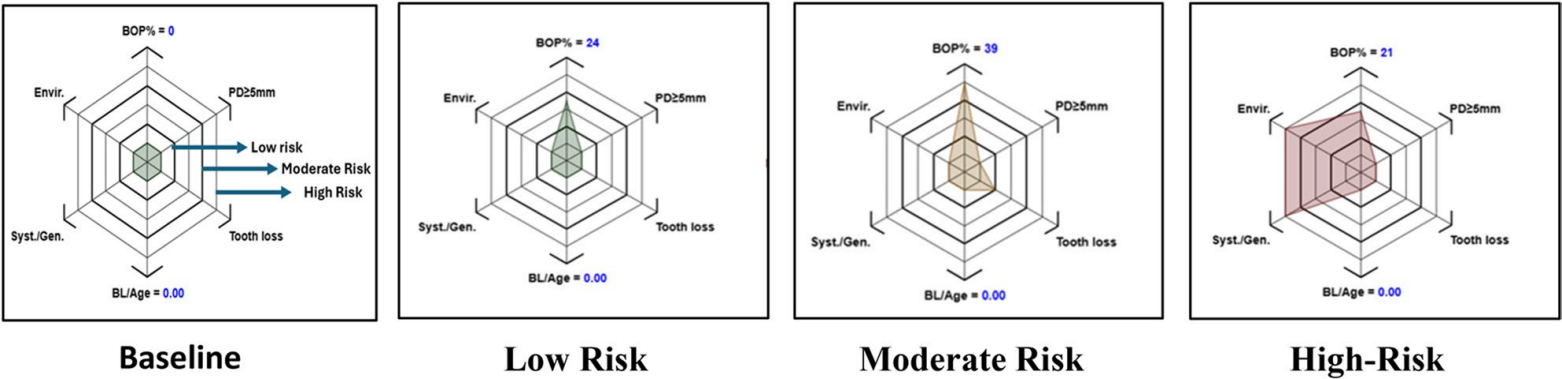
Functional diagram of the PRA model illustrating baseline, low-, moderate-, and high-risk categories

The periodontal risk status of each participant was categorized as low, moderate, or high risk according to the functional diagram of the PRA model developed by Lang and Tonetti (2003), as illustrated in Fig. 2 [19].

Each vector of the polygon represents one risk factor included in the PRA model. For each parameter, three concentric zones indicate increasing levels of risk for periodontal disease progression: the inner zone represents a low risk, the intermediate zone represents a moderate risk, and the outer zone represents a high risk (Fig. 1). All parameters are evaluated simultaneously within the PRA diagram to determine the overall periodontal risk profile. Accordingly, the central area of the polygon corresponds to a relatively low risk of disease progression, whereas values extending beyond the outer ring indicate a high-risk condition. The area located between the two bold rings represents the moderate-risk zone.

According to the PRA model, the risk groups were defined as follows:

- Low-risk group: All parameters are within the low-risk zone of the PRA diagram, or only one parameter extends into the moderate-risk zone.
- Moderate-risk group: At least two parameters were located within the moderate-risk zone, with no more than one parameter extending into the high-risk zone.
- High-risk group: Two or more parameters were extended to the high-risk zone.

The PRA diagrams were generated after completion of data collection using the recorded clinical and behavioral parameters, rather than being calculated chairside during the clinical examination.

After the PRA categories were determined according to the original PRA framework, their associations with other clinical periodontal variables were evaluated. The parameters used to construct the PRA diagram were not included as independent variables in these analyses.

### Statistical analysis

All statistical analyses were performed using IBM SPSS Statistics software (Version 27.0; SPSS Inc., Chicago, IL, USA). Categorical variables are presented as frequencies and percentages (%), and Chi-square tests were used to assess differences between groups for these variables. The distribution of continuous variables was evaluated using the Kolmogorov–Smirnov test and reported as the mean ± standard deviation. Depending on the distribution characteristics, independent samples t-tests or Mann–Whitney U tests were applied to compare continuous variables between groups.

For analytical convenience and modeling purposes, the PRA outcome variable was dichotomized. Accordingly, individuals classified as low risk were coded as 0, whereas those classified as moderate or high risk were coded as 1, with the low risk group serving as the reference category. To identify factors associated with an increased likelihood of being classified as high PRA risk, binary logistic regression analysis was planned. Initially, univariable logistic regression models were constructed for each independent variable. Variables with a *p*-value < 0.25 in univariable analyses were subsequently included in the multivariable model. The multivariable model was constructed using a hierarchical approach, with a retention threshold set at *p* < 0.05. Results were reported as odds ratios (ORs) with 95% confidence intervals (CIs).

The individual components of the PRA were not entered separately into the regression analyses. Probing depth (PD) was included as an independent variable because of its clinical relevance, whereas other PRA parameters were excluded to minimize the risk of multicollinearity and overfitting.

To assess intra-examiner reliability, repeated measurements were performed in 25% of randomly selected participants at a two-week interval. Reliability was evaluated according to the type of variable. For continuous variables (PPD), intra-class correlation coefficients (ICCs) were calculated. For categorical variables (PI and GI), Cohen’s kappa coefficients were used to determine the level of agreement. The level of statistical significance was set at *p* < 0.05.

## Results

Repeated measurements demonstrated high intra-examiner reliability. The intraclass correlation coefficient for probing depth was 0.789. Agreement for categorical variables was also high, with Cohen’s kappa coefficients of 0.810 for plaque index and 0.901 for gingival index.

The descriptive characteristics of the study population are presented in Table 1. The study population consisted of 49% males and 51% females. According to the PRA analysis, 38% of the participants were classified as low risk, 46% as moderate risk, and 16% as high risk. Evaluation of periodontal status revealed that 25% of the participants were periodontally healthy, 43% were diagnosed with gingivitis, and 32% with periodontitis.

**Table 1:** Descriptive characteristics of the study participants

		Sample (n)	Percentage (%)
Gender	Male	49	49
	Female	51	51
Dental Treatment History	No Previous Dental Treatment	40	40
	Previous Dental Treatment	60	60
Marital Status	Single	54	54
	Married	46	46
Educational Level	High School or Lower	10	10
	University Degree or Higher	89	89
Periodontal Status	Periodontally Healthy	25	25
	Gingivitis	43	43
	Periodontitis	32	32
Toothbrushing Habit	Regular	48	48
	Irregular	52	52
PRA Category	Low	38	38
	Moderate	46	46
	High	16	16

### Findings related to PRA

The associations between the PRA risk categories and patient-related factors are presented in Table 2. According to the PRA classification, 62% of the participants were categorized as being in the risk group. Gender-based analysis revealed that, 38% of males and 24% of females were classified as high risk. A statistically significant association was observed between gender and PRA risk status, indicating a higher risk profile among males (*p* = 0.002). No statistically significant differences were observed between male and female participants in terms of age, marital status, educational level, toothbrushing habits, or dental treatment history (*p* > 0.05). Similarly, no significant differences were found between the sexes regarding periodontal parameters, including PI, GI, PPD, and BOP (*p >* 0.05). However, smoking status differed significantly between males and females (*p* < 0.05).

**Table 2:** Association between PRA risk categories and patient-related factors

		High Risk	Low Risk	p*
Gender	Male	38	11	**0.002**
	Female	24	27	
Toothbrushing habit	Irregular	33	19	0.754
	Regular	29	19	
Periodontal status	Periodontally healthy	5	20	**0.001**
	Gingivitis	32	11	
	Periodontitis	32	7	
Marital status	Single	37	17	0.146
	Married	25	21	
Educational level	High School or Lower	9	1	0.052
	University Degree or Higher	52	37	
Dental Treatment History	No Previous Dental Treatment	34	26	0.178
	Previous Dental Treatment	28	12	

^*^: Pearson Chi-square test

Regarding periodontal status, within the risk group showed that 5% of the participants were periodontally healthy, while 32% were diagnosed with gingivitis, and 32% with periodontitis. A statistically significant association was observed between periodontal status and PRA risk classification (*p* = 0.001).

No statistically significant associations were detected between PRA risk status and toothbrushing habits, marital status, educational level, or dental treatment history (*p* > 0.05).

A statistically significant difference was observed between the high and low risk PRA groups in terms of PI and GI (*p* < 0.05). In contrast, the mean age of the participants did not differ significantly between the two PRA groups (*p* > 0.05). The comparative analysis of periodontal parameters across PRA categories is summarized in Table 3.

**Table 3:** Comparison of age and periodontal parameters between PRA risk groups

	High Risk				Low Risk			
	Mean ± SD	Median	Min-Max		Mean ± SD	Median	Min-Max	*p* ^*^
Age	26.84 ± 5.02	27	18–35		27.74 ± 5.56	28	19–35	0.407
Plaque Index	1.80 ± 0.65	1.7	0.35-3		0.92 ± 0.67	0.94	0-2.07	**0.001**
Gingival Index	1.72 ± 0.64	1.67	0.32-3		0.74 ± 0.58	0.96	0-1.60	**0.001**

^*^ Mann Whitney U test

The binary logistic regression model evaluating the association between PRA and demographic variables (Model I) was statistically significant (*p* < 0.05). Gender was significantly associated with PRA status, with male participants exhibiting a 3.89-fold higher likelihood of belonging to the PRA risk group compared with females. In the multivariable logistic regression model, male gender remained an independent predictor, with a 4.30-fold increased risk relative to females. Educational level was also significantly associated with PRA, as participants with a lower educational level demonstrated a 6.40-fold higher PRA risk compared with those with a higher educational level.

The regression model assessing the relationship between PRA and periodontal parameters (Model II) was likewise statistically significant (*p* < 0.05). Full-mouth PI and GI scores differed significantly across PRA categories. Participants in the high-risk PRA group exhibited 7.12 fold higher PI values and 13.61-fold higher GI values compared with those in the low-risk group. In the multivariable logistic regression analysis, an increase in PI was associated with a 5.58-fold increase in PRA risk.

Periodontal health status was also associated with PRA risk, with the likelihood of gingivitis and periodontitis being 11.64-fold and 14.29-fold higher, respectively, compared with periodontally healthy individuals. Additionally, increasing probing pocket depth was strongly associated with PRA risk, corresponding to a 30.15-fold increase in the likelihood of belonging to the PRA risk group.

The results of the binary logistic regression analyses examining the associations between PRA and participants’ demographic and periodontal parameters are presented in Table 4.

**Table 4:** Logistic regression models for factors associated with PRA

		Univariate Logistic Regression			Multivariate Logistic Regression		
		OR	95% CI	*p*	OR	95% CI	*p*
MODEL I (Demographic Parameters) *p* < 0.001							
Age		0.97	0.89–1.04	0.40			
Educational level Ref: High	Low	6.40	0.77–52.74	0.008*			
Marital status Ref: Married	Single	1.83	0.80–4.13	0.15			
Gender Ref: Female	Male	3.89	1.63–9.25	0.002*	3.44	1.39–8.51	0.007*
Dental Treatment History Ref: No previous dental treatment	Previous Dental Treatment	1.80	0.76–4.16	0.18			
Toothbrushing habit Ref: Irregular	Regular	1.14	0.50–2.55	0.75			
MODEL II (Periodontal Parameters) *p* < 0.001							
Periodontal status Ref: Periodontally healthy	Gingivitis	11.64	3.52–38.46	< 0.001			
	Periodontitis	14.29	3.93–51.87	< 0.001			
Plaque Index		7.12	3.12–16.24	< 0.001*	7.12	3.12–16.24	0.001*
Gingival Index		13.61	4.69–39.45	< 0.001*			
Probing Pocket Depth		36.15	6.42-203.42	< 0.001			

*OR* Odds Ratio, 95% *CI* 95% Confidence Interval

* *p* < 0.05, statistically significant

## Discussion

In this cross-sectional pilot study, periodontal risk profiles of young adults were assessed using the Periodontal Risk Assessment model, and the distribution of PRA risk categories together with their associations with selected demographic and clinical variables were examined. Within the study population, participants were distributed across low, moderate, and high PRA risk groups, indicating heterogeneity in periodontal risk profiles among young adults. According to the PRA model, it was observed that not only individuals diagnosed with gingivitis or periodontitis but also those who were clinically periodontally healthy could be classified within the high-risk category. **I**n the present study, a considerable proportion of participants were classified within the PRA risk group, with 62% of individuals categorized as having moderate or high periodontal risk. Among the evaluated factors, male sex was associated with a higher likelihood of belonging to the PRA risk group. In addition, higher plaque index and gingival index values were observed among individuals classified in the high-risk PRA category.

Global epidemiological evidence has shown that the prevalence of periodontal diseases among young adults increased between 1990 and 2019, which may reflect a growing exposure to risk factors associated with periodontal disease [31]. The identification of periodontal risk in young populations is particularly important, as periodontal diseases often develop gradually over time and may remain clinically silent during the early stages [31, 32]. For individuals, understanding the relationship between periodontal disease and its risk factors may encourage young adults to seek regular dental examinations, particularly when one or more risk factors are present [33]. Increased awareness of these risk factors and of the importance of oral health may also promote better oral hygiene practices and healthier lifestyle choices, which may help prevent or delay the onset of periodontitis.

In the present study, the observation that more than half of the study population was classified within the moderate or high periodontal risk categories represents a notable finding. This distribution of risk cannot be explained solely by conventional clinical periodontal parameters used in the diagnosis of periodontal disease, such as probing pocket depth, bleeding on probing, and radiographic bone loss. It may also reflect the influence of behavioral and systemic factors involved in periodontal disease development, including smoking and the presence of systemic conditions.

This finding suggests that even individuals presenting with relatively favorable clinical periodontal parameters may still exhibit a potential susceptibility to future periodontal disease when certain risk factors are present. Therefore, the assessment of periodontal disease should consider not only current clinical findings but also individual risk factors. In this context, the PRA model, which integrates conventional diagnostic parameters with individual risk indicators, may provide a more comprehensive framework for periodontal evaluation. Such an approach may be useful not only for assessing the current disease status but also for planning individualized preventive strategies and maintenance intervals. Furthermore, given the increasing prevalence of periodontal disease with age, identifying risk distributions in young populations is crucial for guiding early intervention strategies and informing population-based oral health policies [19].

Previous epidemiological studies have generally focused on the prevalence of periodontal diseases and the risk factors associated with them [34, 35]. For example, a national-level study conducted in Egypt and another study performed in a Portuguese population reported that periodontitis was associated with several demographic and behavioral factors, including male sex, age, educational level, and oral hygiene habits [34]. However, such studies mainly describe the distribution of existing periodontal destruction within a population and may not fully capture individuals who have not yet developed evident periodontal breakdown but possess a high-risk profile. In this context, the PRA-based risk assessment approach used in the present study may provide a complementary evaluation framework by enabling the identification of risk profiles among individuals with different periodontal conditions, thereby facilitating earlier recognition of periodontal disease susceptibility and the planning of individualized preventive strategies.

PRA analyses showed that male participants exhibited a significantly higher periodontal disease risk than females, in agreement with previous studies reporting greater susceptibility to periodontal diseases among men [35, 36]. This pattern may be related to factors such as a higher prevalence of smoking, lower adherence to routine dental visits, and poorer oral hygiene practices [37–39]. Additionally, unmarried individuals demonstrated a higher periodontal risk compared with married participants, suggesting that marital status may serve as a potential social determinant of periodontal health in young adults [40]. The significant associations observed between high PRA risk and full-mouth plaque index and gingival index values are also consistent with the existing literature [41–43]. In a systematic review and meta-analysis conducted by Lertpimonchai et al. in 2017, a strong relationship was reported between plaque accumulation and the development of periodontal disease, with similar evidence supporting the association between gingival index scores and periodontal outcomes [41–43]. In line with these findings, our study demonstrated that individuals with elevated full-mouth plaque index values exhibited a 5.58-fold increased periodontal risk. Collectively, these results suggest that gender, marital status, and oral hygiene indicators may be associated with periodontal risk patterns among young adults. In this study, certain variables such as probing pocket depth and periodontal status (gingivitis or periodontitis), which may be indirectly related to PRA, were evaluated only in univariate analyses. However, considering that these parameters may overlap with some components of the PRA model, caution should be exercised when interpreting the observed associations. Therefore, these variables were not included in the multivariable analysis model, and the univariate analyses were conducted using an exploratory approach to examine their relationship with PRA risk categories.

Periodontal risk assessment is currently used to identify individuals at high risk for disease development during the initial examination and to plan supportive periodontal care programs following active treatment [44]. Accordingly, the PRA model introduced by Lang and Tonetti in 2003 was designed to enable a comprehensive, patient-level evaluation of periodontal risk independent of diagnostic categories [19]. According to the updated periodontal disease classification, periodontitis is assessed using a staging and grading framework, whereas the diagnosis of gingivitis is largely based on threshold values of bleeding on probing. Although supportive clinical measurements are recorded, their integration into a unified risk profile often relies on clinician judgment or existing guidelines. In contrast, risk-based scoring systems such as PRA facilitate the synthesis of clinical, environmental, and genetic risk factors into a personalized risk model [6]. Consistent with the multifactorial structure of the PRA model, a small number of participants who were classified as periodontally healthy were nevertheless categorized as high-risk. This finding was primarily driven by unfavorable environmental and systemic risk indicators, particularly smoking status and systemic conditions, which contributed to elevated PRA scores despite the absence of clinical periodontal destruction. This observation highlights that PRA can help identify individuals who could potentially be at increased risk for future periodontal disease. It should also be emphasized that the risk-based approach used in this study is not intended to replace the existing diagnostic classifications of periodontal diseases. Rather, periodontal risk assessment aims to serve as a complementary tool to current diagnostic systems by helping clinicians identify individuals who may be more susceptible to disease development or progression [44, 45]. In this respect, the PRA approach may contribute to the evaluation of periodontal risk profiles, particularly in young populations, and support the consideration of individual risk factors in clinical practice.

However, the findings of the present study should be interpreted with caution in light of the study design and sample characteristics. Nevertheless, several limitations of the present study should be acknowledged. The restriction of the sample to a young adult population and recruitment from a university dental clinic may limit the generalizability of the findings to older age groups and the broader population. Moreover, the relatively limited number of comparative studies evaluating periodontal risk using PRA complicates broader contextualization of the results. Another limitation is the inability to fully account for site-specific local factors, such as anatomical variations, localized plaque-retentive areas, or interindividual differences in biological response, which may influence periodontal risk at the individual level. Since this study was designed as a cross-sectional investigation, longitudinal follow-up was not performed to evaluate changes in periodontal status over time. Therefore, the predictive validity of PRA risk categories could not be assessed within the scope of the present study. Future multicenter longitudinal studies including broader age ranges and heterogeneous risk profiles may provide more robust evidence regarding the applicability of the PRA model across different populations.

## Conclusion

Within the limitations of this cross-sectional pilot study, the following conclusions can be drawn:

- Periodontal risk was found to be highly prevalent among young adults. According to PRA analysis, more than half of the participants were classified as being at risk, indicating that periodontal risk in young individuals is clinically significant.
- Male sex, increased plaque index, gingival index, and probing pocket depth were identified as the factors most strongly associated with an increased periodontal risk.
- Individuals diagnosed with gingivitis and periodontitis exhibited significantly higher PRA risk levels compared with periodontally healthy individuals.

These findings support the use of PRA not only in patients with established periodontitis but also as a practical and valuable tool for early risk identification in young adults with gingivitis, enabling timely preventive and risk-based periodontal care. Since the parameters used in the PRA model are largely derived from routine periodontal examination findings, the tool can be easily incorporated into daily clinical practice and may assist general dentists in rapidly identifying patients at increased periodontal risk.

## Supplementary Information

Supplementary Material 1.

## Data Availability

Data are available from the authors upon reasonable request.

## Abbreviations

PRA: Periodontal Risk Assessment
AAP: American Academy Of Periodontology
EFP: European Federation Of Periodontology
BOP: Bleeding On Probing
PPD: Periodontal Probing Depth
NS: Non-Smokers
FS: Former Smokers
OS: Occasional Smokers
MS: Moderate Smokers
HS: Heavy Smokers
PI: Plaque Index
GI: Gingival Index
RBL: Radiographic bone loss
OR: Odds Ratio
ICC: İntra-Class Correlation Coefficients
BL/Age: Bone Loss/Age
